# Wearable technology in the management of complex chronic illness: preliminary survey results on self-reported outcomes

**DOI:** 10.3389/fdgth.2025.1662255

**Published:** 2025-10-08

**Authors:** Abbey Sawyer, Rory Preston, Harry Leeming, Luke Martin-Fuller, Amy Proal, David Putrino

**Affiliations:** ^1^ Cohen Center for Recovery from Complex Chronic Illness, Icahn School of Medicine at Mount Sinai, New York, NY, United States; ^2^ Visible Health Inc., Wilmington, DE, United States; ^3^ Polybio Research Foundation, Medford, MA, United States

**Keywords:** long covid, myalgic encephalitis, home monitoring, ME/CFS, survey

## Abstract

**Introduction:**

Complex chronic illnesses like Long Covid (LC) and Myalgic Encephalomyelitis/Chronic Fatigue Syndrome (ME/CFS) are marked by fluctuating symptoms, often exacerbated by physical, cognitive, or emotional exertion in a phenomenon known as post-exertional malaise (PEM). Home monitoring technologies offer potential benefits by enabling individuals to track symptoms and biometrics, aiding in disease self-management. However, the general effectiveness of such tools is still unknown.

**Methods:**

A random sample of users of the Visible mobile application (Visible Plus; requires both the armband and paid subscription), aged 18 or older and with self-identified complex chronic illnesses such as LC or ME/CFS, were invited to complete an online survey regarding the impact of the app on their chronic disease self-management. Descriptive statistics related to the responses were analyzed and reported.

**Results:**

The survey was distributed to 2,636 people, with 1,301 participants responding (49.3% response rate). The average age was 46 years. 82% of respondents were female, 8% were male, 8% were non-binary, and 2% preferred not to say or preferred to self-describe. Participants self-identified as having ME/CFS only (*n* = 534, 42%), LC only (*n* = 396, 31%), ME/CFS and LC (*n* = 236, 18%), or another illness (*n* = 122, 10%). Of the *n* = 2,636 randomly selected subscribers, the mostly commonly listed “other illnesses” were Postural Orthostatic Tachycardia Syndrome (POTS, 6%), fibromyalgia (5.2%), Ehlers Danlos Syndrome (EDS; 1.7%) and Mast Cell Activation Syndrome (MCAS, 1.2%). Of those with at least 30 days of data, 77% reported seeing an improvements associated with app use, corresponding to 23% of all invited users, 85% (corresponding to 29% of all invited users) reported feeling somewhat (53%) or significantly (32%), and 94% (corresponding to 33% of all invited users) reported a better understanding of their energy budget.

**Discussion:**

Home-monitoring based mobile applications are feasible and acceptable for a motivated subgroup of people with energy-limiting complex chronic illnesses, and are associated with self-reported benefits in energy management and participation in daily activities. The findings of this study should be interpreted as descriptive and hypothesis-generating and do not represent clinically significant effects, underscoring the need for randomized controlled trials to formally evaluate efficacy. Future studies should incorporate a comparison group to better differentiate intervention effects from improvements gained through lived experience.

## Introduction

Complex chronic illnesses such as Long Covid (LC) and Myalgic Encephalomyelitis/Chronic Fatigue Syndrome (ME/CFS) often involve symptoms that can fluctuate over time. For some people, this may mean feeling relatively well on one day, followed by a rapid onset or worsening of symptoms the next (e.g., fatigue, cognitive impairment, headache, shortness of breath), though patterns can vary between individuals. At times, the onset of these worsening symptoms may have a clear cause, for example, an increase in exertion, which is often referred to as post-exertional malaise (PEM) and is a feature of complex illnesses such as LC and ME/CFS. Furthermore, different types of exertion can trigger PEM, including but not limited to physical, cognitive, emotional, social forms of exertion (1, 2). At other times, the underlying cause behind the worsening of symptoms can be uncertain. Similarly, some people report a rapid onset of initial symptoms (e.g., at the start of the illness), whereas others note a more progressive increase in impairment (3).

The benefits of home-monitoring in health and disease are well understood (4). The efficacy of daily monitoring has been established in improving health in people living with chronic obstructive pulmonary disease (5), cystic fibrosis (6), cardiovascular disease (7), high health-risk older adults managing multiple pathologies (8), and healthy individuals (9). While there are few reports of home monitoring systems that allow people with energy-limiting complex chronic illnesses to track and report symptoms (10), self-monitoring and reporting of symptoms may provide this group with actionable feedback regarding various aspects of disease self-management. For example, heart rate (HR) monitoring may help people with ME/CFS understand and manage PEM symptoms and support participation in activities of daily living (11). The benefits of these self-monitoring strategies may include early identification of PEM that may impact on physical functioning (12), and access to data that can guide individualized care and inform treatment decisions in collaboration with a clinical team (11). Overall, applying home monitoring technology in the context of energy-limiting complex chronic illness has the potential to improve fatigue management, emotional wellbeing, and confidence in self-management (13).

The Visible application (Visible Health Inc., Delaware, USA; https://makevisible.com/) is a real-world mobile application that provides people with LC, ME/CFS and other energy-limiting conditions the ability to self-monitor symptoms and biometrics. The impact of using the application with a wearable heart monitor to record biometrics to manage illness, energy budgets, symptoms and activity has not been evaluated.

### Aims

In people self-identifying with complex chronic illness using a mobile application connected to a wearable device to monitor biometrics and symptoms, we aimed to describe the participant characteristics and outcome results of an application-wide survey.

## Methods

An online survey was distributed to users of the Visible mobile application (Visible Health Inc., Delaware, USA), who had been using the app in conjunction with a Polar Verity Sense™ HR monitor (Polar Electro, Inc., Kempele, Finland) in April 2024. The Polar Verity Sense™ armband is a wearable device that uses an optical sensor to continuously measure HR and was selected due to its comfort, ease of use and accuracy. Participants were using the Visible Plus version, which differs from the free version of the app in that it requires both the armband and an active subscription.

The survey was designed by the investigators to assess the participants’ experiences, specifically focusing on the perceived impact of their use of the Visible app and armband on PEM episodes. Survey questions covered multiple dimensions of PEM, including fatigue, pain, cognitive function, and other self-reported health outcomes, aiming to capture changes over time as perceived by the users (Table 1). Before distribution, the survey underwent pilot testing amongst the research team solely for technical validation of the survey.

**Table 1:** Visible online survey questions.

Description	Response Options
Q1. Since using visible with the armband, my feeling of control over my illness has.	Significantly improved
Q2. Since using visible with the armband, my sustainable physical activity levels have.	Somewhat improved
Q3. Since using visible with the armband, my understanding of my energy budget has.	Neither improved nor worsened
Q4. Since using visible with the armband, the frequency of my PEM episodes and/or symptom flares has.	Somewhat worsened
Q5. Since using visible with the armband, the severity of my PEM episodes and/or symptom flares has.	Significantly worsened
Q6. Since using visible with the armband, the amount of activity I can perform without triggering PEM episodes and/or symptom flares has.	
Q7. Since using visible with the armband, my ability to manage my energy effectively has.	
Q8. Since using visible with the armband, my ability to take part in daily activities & things I want to do has.	

Eligibility for survey participation required that subscribers of the Visible app had logged a minimum of 30 days of wearable HR data. This threshold was set to ensure that participants had ample data for assessing trends or changes in PEM symptoms. However, former subscribers who had stopped collecting wearable data were still eligible to participate, provided they met the criterion of 30 recorded days during their subscription period.

Participants were provided with clear information about the purpose of the survey, its voluntary nature, and the assurance of anonymity at the start of the survey by Visible Health. Participants provided electronic consent prior to enrollment. By clicking an “I accept” option, they agreed to allow Visible to collect and use their anonymous data for research purposes. Only anonymized, aggregate data were analyzed and reported in this study. Further information about the ethical processes is detailed in the ‘Ethical Approval and Consent to Participate’ section of this manuscript.

**Figure 1:**
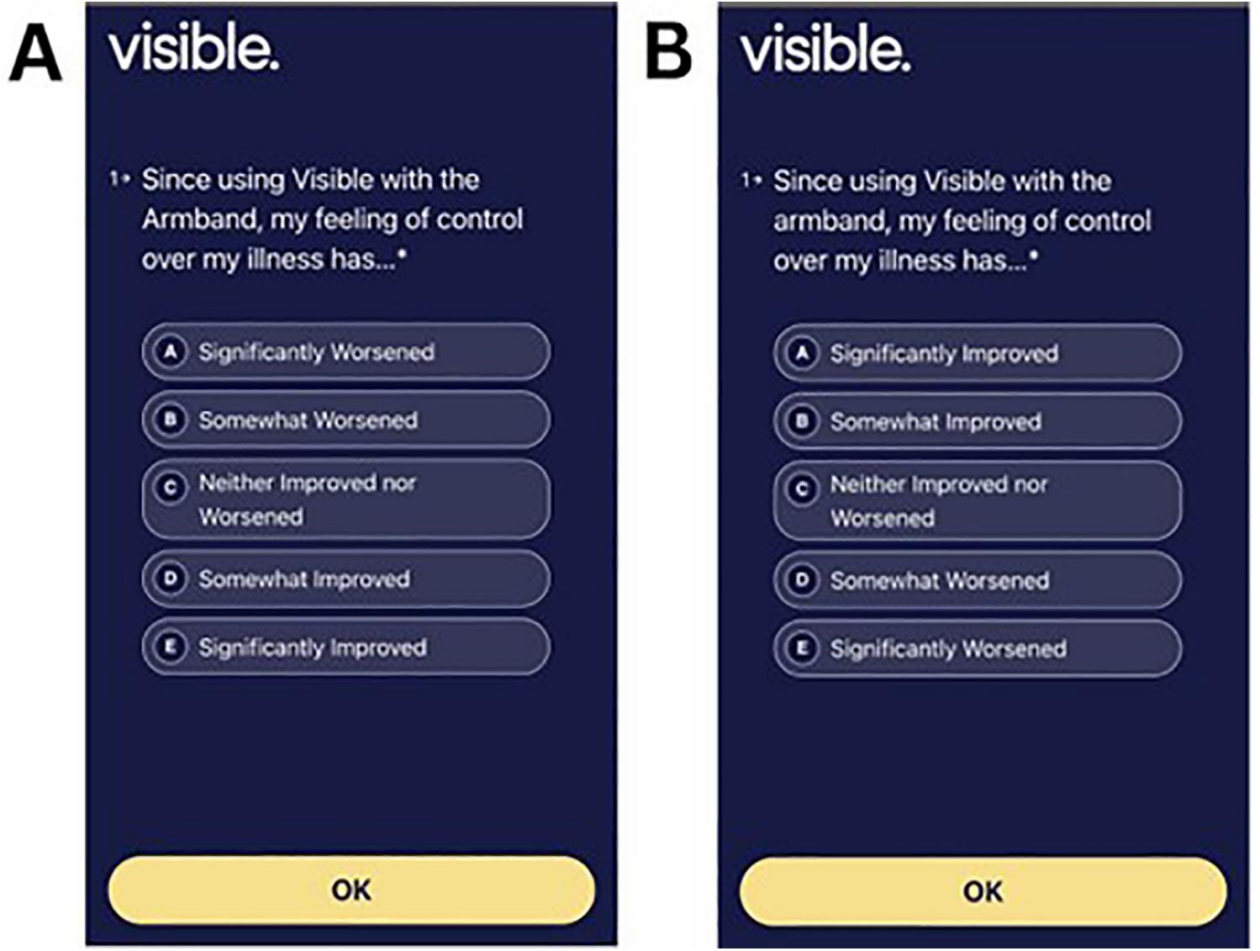
Participants were randomly presented with survey responses to the questions in Table 1 with more favorable responses in ascending **(A)** or descending **(B)** order. Screenshots from: Visible app, https://www.makevisible.com/.

The survey was distributed electronically via a link embedded within the Visible mobile app and email invitations. To minimize response bias, the order of answer choices was randomized, with options presented in either ascending or descending order for each participant (Figure 1).

### Participants

People aged ≥18 years with self-identified complex chronic illness such as LC, ME/CFS, or people experiencing other causes of energy limitation who were using the Visible application and opted to share their data anonymously were eligible for inclusion.

### Analysis

Descriptive statistics were used to summarize data.

## Results

### Participant demographics

The survey was circulated to *n* = 2,636 randomly selected subscribers from a total pool of *n* = 4,537 subscribers, yielding a total of *n* = 1,301 responses (49.3% response rate). The mean ± standard deviation age of participants was 46 ± 12 years. 82% of respondents indicated that their gender was female, 8% were male, 8% were non-binary, and 2% preferred not to say or preferred to self-describe. Participants self-identified as having ME/CFS only (*n* = 534, 42%), LC only (*n* = 396, 31%), ME/CFS and LC (*n* = 236, 18%), or another illness (*n* = 122, 10%). Of the *n* = 2,636 randomly selected subscribers, the mostly commonly listed “other illnesses” were Postural Orthostatic Tachycardia Syndrome (POTS, 6%), fibromyalgia (5.2%), Ehlers Danlos Syndrome (EDS; 1.7%) and Mast Cell Activation Syndrome (MCAS, 1.2%).

### Survey responses

**Figure 2:**
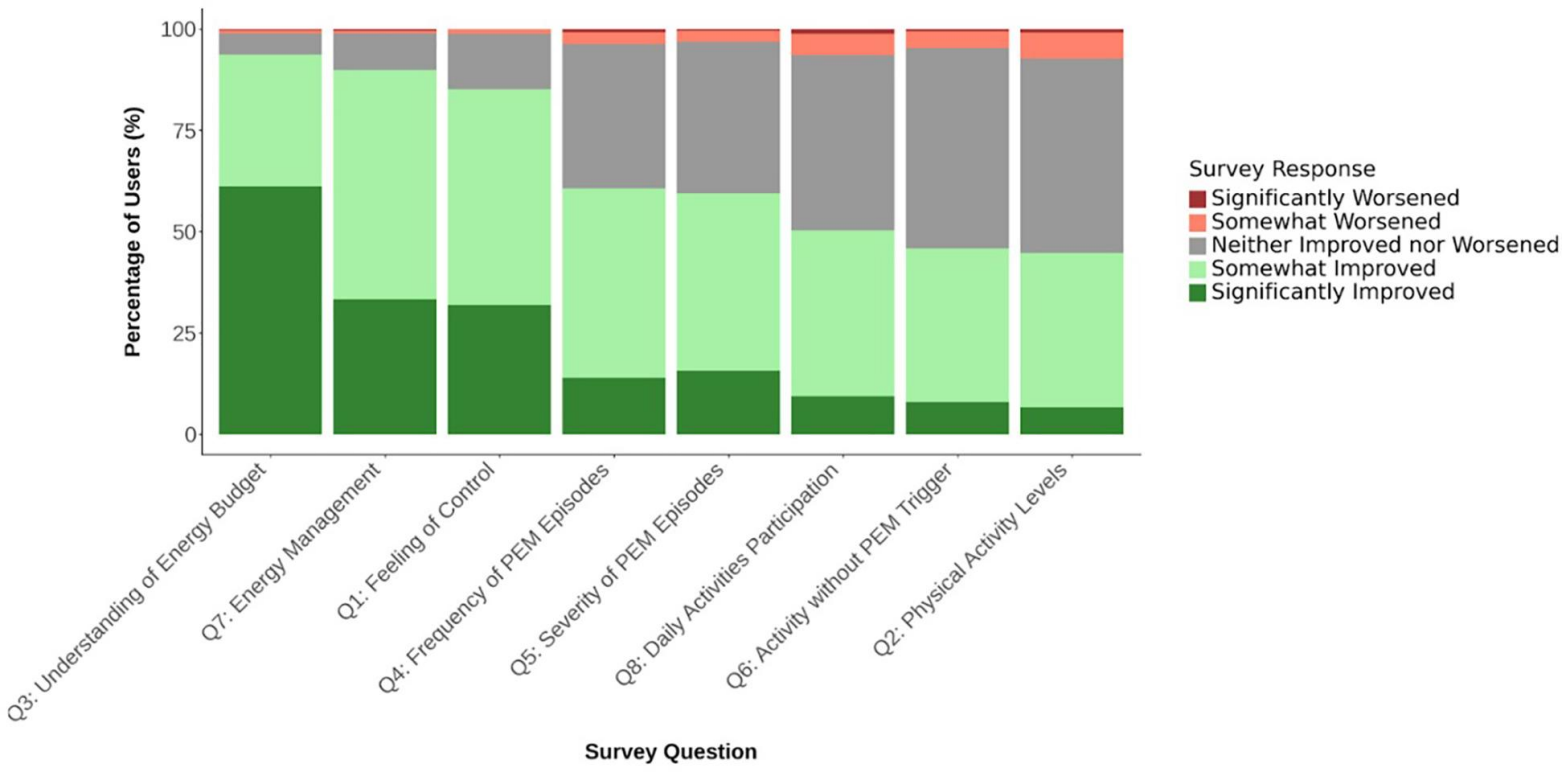
User-reported changes for each survey question with visible armband.

Table 2 highlights the distribution of user responses for users with at least 30 days of activity. In these users, 77% reported seeing an improvements associated with app use (corresponding to 23% all invited users), and 85% (corresponding to 29% of all invited users) reported feeling somewhat (53%) or significantly (32%) more in control of their illness since using Visible with the armband. 94% indicated a better understanding of their energy budget (corresponding to 33% of all invited users), and 90% reported experiencing enhanced energy management (corresponding to 31% of all invited users) (Figure 2). Reductions in the frequency and severity of PEM episodes were noted by 61% and 60% of respondents, respectively (corresponding to 15% of all invited users). Fewer reported substantial changes in physical activity levels (45%; corresponding to 12% of all invited users). There were no statistically significant differences between the distribution of data from 30 + days to 60 + or 90 + days (Supplementary Material 1).

**Table 2:** Distribution of user responses for users with 30+ days of wearable data.

Survey question	Significantly worsened	Somewhat worsened	Neither improved nor worsened	Somewhat improved	Significantly improved	Improved
Q1: Feeling of control	0.08 (1)	1.16 (15)	13.51 (176)	53.34 (694)	31.91 (415)	85.25 (1,109)
Q2: Physical activity levels	0.93 (12)	6.44 (84)	47.67 (620)	38.35 (499)	6.60 (86)	44.95 (585)
Q3: Understanding of energy budget	0.31 (4)	0.78 (10)	5.20 (68)	32.45 (423)	61.26 (797)	93.71 (1,220)
Q4: Frequency of PEM episodes	0.78 (10)	2.95 (38)	35.64 (464)	46.66 (607)	13.98 (182)	60.64 (789)
Q5: Severity of PEM episodes	0.47 (6)	2.72 (35)	37.34 (486)	43.79 (570)	15.68 (204)	59.47 (774)
Q6: Activity without PEM trigger	0.54 (7)	4.19 (54)	49.53 (644)	37.81 (492)	7.92 (103)	45.73 (595)
Q7: Energy management	0.39 (5)	0.70 (9)	9.08 (118)	56.44 (734)	33.39 (434)	89.83 (1,168)
Q8: Daily activities participation	1.16 (15)	5.36 (70)	43.17 (562)	40.99 (534)	9.32 (121)	50.31 (655)

Data are presented as percentages with counts in parentheses. Percentages in this table reflect only survey respondents (*n* = 1,301); % data related to the proportion relative to all invited users (*n* = 2,636) is provided in the main text.

Among users who connected their Armband in March 2024, 86% continued using it 30 days later. For those who connected their Armband in January 2024, 75% were still using it after 90 days.

## Discussion

In a large sample of people with complex chronic illness, the majority of survey respondents reported substantial improvements across most categories. Almost all indicated a better understanding of their energy budget, enhanced energy management and improvements in the feeling of control. Reductions in the frequency and severity of at least one aspect of their PEM was reported by 77% of users, and over half of respondents reported increased participation in daily activities. In line with other work and the incidence of some complex chronic illness (3, 14), the survey respondents were predominantly female. These descriptive findings highlight self-reported benefits of using home-monitoring applications, including the Visible application and armband, and provide preliminary insights into how individuals managing energy-limiting complex chronic illness perceive their use of such tools.

Despite the potential benefits, in the present study, fewer users reported substantial increases in their tolerance of physical activity. A possible explanation is that using the Visible app may increase users’ awareness of activity thresholds that precipitate PEM. Prior to using the app, users may not have recognized that certain routine activities could trigger a response. Adopting a data-informed pacing strategy may help users identify these thresholds, which could contribute to the perception of reduced physical activity capacity in the survey responses. This possible explanation is speculative and not directly measured in the study and as such the findings should be interpreted as descriptive and hypothesis generating. In addition, the findings related to physical activity in the present study may reflect that while the app helps users pace themselves and avoid overexertion and PEM, these strategies do not directly influence the underlying physiological processes. In LC, local and systemic metabolic disturbances, severe exercise-induced myopathy, infiltration of amyloid-containing deposits, and immune cells in skeletal muscles are key characteristics of post-exertional malaise, which likely contribute to limited physical activity tolerance (15). Despite the demonstrated potential of mobile applications for symptom management in complex chronic illness, there still remains a critical need for clinical trials that address the physiological drivers of PEM.

Reduced fatigue following the use of home-monitoring and activity pacing have been reported elsewhere. In a systematic review of 14 studies of at least good quality [PEDro scoring (16)], activity pacing interventions were found to be an effective strategy to reduce fatigue and psychological distress, and to improve physical function in people with ME/CFS (17). In people with LC, simple pacing strategies such as the WHO Borg CR-10 pacing protocol has demonstrated significant reductions in PEM and improvement in activity levels over a 6-week period (18). The current work shows a high level of consistency with the benefits experienced by users of apps that target other chronic illnesses and conditions such as COPD (19), cystic fibrosis (6) and chronic pain (20). Although relatively little has been done to evaluate the impact of mobile applications on energy-limiting complex chronic illness, our results are consistent with others (11, 21). Clague-Baker et al. (11) surveyed 488 people with ME/CFS in 2023 and found that HR monitoring helped 72% of participants better understand their personalized PEM. Other benefits of HR monitoring were listed as having real-time feedback on current or prior activity (69%) and that this type of monitoring helped stop the boom/bust or push-crash cycle that is common to this condition (64%). Despite these perceived benefits, many (57%) still felt that support from medical professionals to utilize the pacing data was lacking (7), highlighting a persistent gap in integrating digital self-monitoring tools into clinical care. This gap may reflect clinicians’ limited familiarity with digital self-monitoring tools, competing demands during appointments, or uncertainty regarding how to incorporate individualized pacing data into care plans. As a result, even effective digital interventions may not achieve their full impact without integrated clinician support, underscoring the need for strategies that connect patient self-management with professional guidance. Although our work did not evaluate a single specific approach to chronic disease self-management, it is further validation that data-informed pacing can be highly beneficial to people living with energy-limiting complex chronic illness.

## Limitations and future directions

The survey response rate suggests potential self-selection bias, as non-respondents may include users who discontinued due to lack of benefit, limiting the generalizability of results. Respondents in this study also self-reported their clinical conditions. As such, the authors have referred to the dataset as being derived from people with complex chronic illness, rather than a specific condition. Future work would benefit from focusing on specific diagnoses that have been confirmed clinically in order to further validate these findings. The study collected limited demographic data including age, gender, illness type, which restricts assessment of the sample's representativeness relative to the broader population. Future research should aim to include more comprehensive demographic information to enhance generalizability.

Approximately half (49.3%) of randomly selected survey recipients responded. While this is a high proportion of recipients for in-app surveys, it could still introduce bias toward users who had a positive experience with the app, highlighting the critical need for a controlled study of the utility of mobile applications for complex chronic illness that was beyond the scope of this initial preliminary analysis.

Understanding the natural progression of self-management skills in complex chronic illnesses is important for contextualizing the potential benefits of the intervention. However, this study was designed to explore feasibility and acceptability, rather than a comparative effectiveness trial. As such, we did not include a control group in the current study. Future studies should incorporate a comparison group to better differentiate intervention effects from improvements gained through lived experience.

Participants used the paid Visible Plus version, which requires purchase of the armband and a subscription (as opposed to the free app version). Subscription status may influence perceptions of value, engagement, and retention, introducing potential ascertainment bias, and the results may reflect a subgroup of highly motivated and engaged users. Furthermore, our analysis includes only participants with at least 30 days of use, potentially excluding early discontinuers and biasing results toward more engaged users. Participants who discontinued before 30 days were excluded and unmeasured factors, such as motivation, symptom severity, or type, may have influenced the likelihood of continued use. These factors may limit the generalizability of the findings.

For this initial exploratory survey, self-reported diagnoses were grouped together for pragmatic reasons. However, this approach limits the interpretability of the findings for any individual condition.

Finally, we acknowledge that relying on self-reported symptom and PEM changes without objective activity data is a limitation of the study.

## Conclusion

The results of this descriptive study, conducted in the largest known group of people with energy-limiting complex chronic illness, highlights the feasibility and acceptability of the Visible app for insights regarding their energy-limiting complex chronic illness in a group of motivated users. These self-reported findings are preliminary and hypothesis generating; randomized controlled trials of app-based services for complex chronic illnesses could further validate the findings.

## Data Availability

The raw data supporting the conclusions of this article will be made available by the authors, without undue reservation.
